# Optimal loading dose for the initiation of warfarin: a systematic review

**DOI:** 10.1186/1471-2261-10-18

**Published:** 2010-04-19

**Authors:** Carl Heneghan, Sally Tyndel, Clare Bankhead, Yi Wan, David Keeling, Rafael Perera, Alison Ward

**Affiliations:** 1 Department of Primary Health Care, University of Oxford, Oxford, UK; 2 Department of Health Statistics Fourth Military Medical University, Xi'an, China; 3 Department of Haematology, Oxford Radcliffe Hospitals, Oxford, UK

## Abstract

**Background:**

Selection of the right warfarin dose at the outset of treatment is not straightforward, and current evidence is lacking to determine the optimal strategy for initiation of therapy.

**Methods:**

We included randomized controlled trials in patients commencing anticoagulation with warfarin, comparing different loading dose or different regimens.

We searched Medline, EMBASE, the Cochrane Library and the NHS Health Economics Database up to June 2009. Primary outcomes were time to stable INR and adverse events. We summarised results as proportion of INRs in range from date of initiation and compared dichotomous outcomes using relative risks (RR) and calculated 95% confidence intervals (CIs).

**Results:**

We included 11 studies of 1,340 patients newly initiated on warfarin. In two studies that used single INR measures, a loading dose of 10 mg compared to 5 mg led to more patients in range on day five. However, in two studies which measured two consecutive INRs, a loading dose of 10 mg compared to 5 mg did not lead to more patients in range on day five (RR = 0.86, 95% CI, 0.62 to 1.19, p = 0.37). Patients receiving a 2.5 mg initiation does took longer to achieve the therapeutic range, whilst those receiving a calculated initiation dose achieved target range 0.8 days quicker (4.2 days vs. 5 days, p = 0.007). More elderly patients receiving an age adjusted dose achieved a stable INR compared to the Fennerty protocol (48% vs. 22% p = 0.02) and significantly fewer patients on the age adjusted regimens had high out-of-range INRs. Two studies report no significant differences between genotype guided and 5 mg or 10 mg initiation doses and in the one significant genotype study the control group INRs were significantly lower than expected.

**Conclusion:**

Our review findings suggest there is still considerable uncertainty between a 10 mg and a 5 mg loading dose for initiation of warfarin. In the elderly, lower initiation doses or age adjusted doses are more appropriate, leading to less higher INRs. Currently there is insufficient evidence to warrant genotype guided initiation, and adequately powered trials to detect effects on adverse events are currently warranted.

## Background

Oral anticoagulants are effective for the prevention and treatment of thromboembolic events [1-4] and are used in many conditions including deep vein thromboses (DVT), pulmonary emboli (PE), mitral and aortic valve replacements (MVR/AVR) and atrial fibrillation (AF), together with occasional use in patients with heart failure and those with peripheral and cerebral vascular disease. The use of oral anticoagulants such as warfarin has increased substantially over the last 10 years, particularly within the context of an ageing population [5,6].

Although warfarin is an effective antithrombotic agent the therapeutic range is narrow due to the balance between reducing thrombotic events without increasing the risk of bleeding. To monitor therapy, an International Normalized Ratio (INR) between 2.0 and 3.0 is generally accepted for these conditions with the exception of valve replacements where a higher INR between 2.5 and 3.5 is usually recommended [7,8].

Given the wide variation on dose response to warfarin, careful monitoring is required especially in the initiation phase of treatment. Different methods of initiating warfarin aim to establish the therapeutic window efficiently without causing adverse effects. Some conditions (i.e. deep vein thrombosis) require the establishment of effective anticoagulation quickly to reduce harms, reduce concomitant treatments such as heparin, and reduce costs. In other conditions, such as outpatient diagnosis of atrial fibrillation, the time to establish the therapeutic range is not as crucial.

Initial doses of warfarin include 10 mg [9], 5 mg [10], 2.5 mg [11], and, in the elderly, lower doses such as 1 mg [12]. In addition, initiation dosing can occur by using protocols such as Fennerty's, which relies on consecutive daily INRs over the first four days to predict the next day's warfarin dose [13]. There has also been considerable interest in genotype guided warfarin initiation [14]. Warfarin is a racemic mixture of R and S enantiomers; the more potent S-warfarin is metabolised in the liver by cytochrome P450 2C9. The wild-type allele is labelled CYP2C9*1, two other alleles, CYP2C9*2 and CYP2C9*3, result in warfarin being metabolised more slowly, and carriers of these alleles potentially have a greater risk of bleeding during initiation of warfarin and subsequently require lower doses [15].

Balancing the need for effective anticoagulation, with reduced time to therapeutic INR and without concomitant increases in adverse events is important, not only for patients, but for heath care systems in terms of economic costs [16]. Selection of the right warfarin dose at the outset is not straightforward, and current evidence is lacking to determine the optimal strategy; therefore we set out to systematically review the literature on the most effective methods for initiating warfarin.

## Methods

We included randomized controlled trials in patients commencing anticoagulation with warfarin, comparing different loading dose or different regimens at the initiation of therapy. Included trials had one or more of the following outcomes: time to stable INR range, doses withheld, supra-therapeutic INRs and/or, adverse events.

We searched the following databases: Medline (1966 - 2009), EMBASE, the Cochrane Library including the Cochrane Central Register of Controlled Trials (CENTRAL), Database of Reviews of Effectiveness (DARE) and the NHS Health Economics Database up to June 2009 using a maximally sensitive strategy (Dickersin et al 2002). Search terms included Warfarin/ad (Administration & Dosage), Anticoagulants, Warfarin, (anticoagula* or warfarin or coumadin).tw. combined with the following search terms: Drug Administration Schedule, Dose-Response Relationship, Drug, ((Fennerty* or age adjusted or empirical or fixed) adj5 (regime* or method* or protocol* or algorithm*)).tw., ((dosing or dose* or dosage*) adj5 (empirical or regime* or method* or protocol* or algorithm* or nomogram*)).tw. ((initial or initiation or induction or loading) adj3 (dose* or dosage* or dosing)).tw.

Two researchers independently reviewed the title and abstracts of electronic searches, obtaining full-text articles to assess inclusion where necessary. We performed citation searches and reviewed references of all full text papers retrieved. Disagreements were resolved by discussion with a third author. Where data was insufficiently reported in the published paper we wrote to the original authors for clarification and further information.

### Data extraction

Two authors (ST and CB) independently extracted data and assessed quality with an extraction template; disagreements were documented and resolved by discussion with a third author (CH). Primary outcomes were time to stable INR; supra-therapeutic INR, sub-therapeutic INRs, Vitamin K given, and the following serious adverse events: bleeding, thrombotic events and death. Secondary outcomes sought were economic costs, hospital stay, length of concomitant therapy and frequency of INR tests. To define adverse events we used the definitions reported in the primary studies (see Table 1).

**Table 1:** Study Characteristics

Study	Recruitment Setting	Eligible Population (average age)	Inclusion/Exclusion criteria	Protocol Outcomes
**5 mg vs. 10 mg**				

Harrison (1997) Canada	Inpatients & outpatients	51 patients * (64 yrs) %not available	INR target 2.0-3.0	1)Time to INR in range, 2) INR >3.0, 3) Time to reduction in factors II, X and protein C
Crowther (1999) Canada	Thromboembolism unit	53 patients * (65 yrs) 47% male	INR target 2.0-3.0. Exclusions: contraindication to warfarin or geographically inaccessible	1) Proportion with INR in range for 2 consecutive days on days 3 & 4, or 4 & 5 and INR not >3.0
Kovacs (2003) Canada	Outpatient clinics	201 patients with DVT or PE (55 yrs) 56% male	Exclusions: baseline INR >1.4, thrombocytopenia, <18 yrs, hospitalised, oat in previous 2 wks, high risk of bleeding	Primary Outcome: 1) Time to INR >1.9. Secondary Outcomes: 2) INR in-range by day 5 3) VTE by day 90 4) Major bleeding by 28 days 5)INR >5.0 6)Number of INRs in 28 days 7) Death by 90 dys
Quiroz (2006) USA	Inpatients	50 patients with DVT or PE (50 yrs) 54% male	Exclusions: <18 yrs, not available for clinic f/up, warfarin or heparin >36 hrs, creatinine clearances of <30 ml/min, life expectancy <3 mths, high risk of bleeding	Primary Outcome: 1) Time to INR >1.9 on 2 consec. days. Secondary Outcomes: 2) Recurrent VTE at 14 days 3) Death at 14 days 4) Major bleeding at 14 days 5) INR >5 at 14 days
**5 mg vs. 2.5 mg**				

Ageno (2001) Canada &Italy	Inpatients	232 patients with heart valve replacement (64 yrs) 56% male	INR target 2.0 (range 1.5-2.6). Exclusions: baseline INR >1.3	Primary Outcome: 1) % INR >2.6 Secondary Outcomes: 2) Time to INR in range 3) % out of range 4) Vit. K/bleeding/thromboembolic events 5) Dose adjustments/mean daily dose
**5 mg vs. calculated dose**				

Shine (2003) USA	Inpatients	90 patients with AF, DVT, PE or other (61 yrs) 58% male	INR target 2.0-3.0 & INR = 1.4. Exclusion; warfarin in previous 3 months	1)Time to INR in range 2) Hours in hospital 3) Number with INR 2.0-3.0 4) Factor II protein activity 5) Clinical complications 6) INR ever >4.0 or a rise of 2.0 over 2 days to >3.0
**Age adjusted**				

Roberts (1999) Australia	Inpatients	65 patients with AF, DVT, PE & other (74 yrs) 70% male	INR target 2.0-3.0. Exclusions: prolonged diarrhoea, nasogastric/enteral feeds, commencing amiodarone, advanced malignancy, Vitamin K in previous 2 wks	Primary Outcome 1)Time to INR in range on 2 consecutive days or if previous day within 0.5 Secondary Outcomes 2) No. with INR >4.0 in first week 3) Dose withheld in wk1 4) No. of days with heparin
Gedge (2000) UK	Inpatients	127 patients with - AF, DVT, PE & other (75 yrs) 50% male	INR target 2.0-3.0. Elderly patients with standard indications.	1)Time to INR >2.0 2) Days INR in range 3)Number with INR >4.5 4) Dose prediction day 4

**Genotyping**				

Hillman (2005) USA	Inpatient & Outpatients	38 with DVT, PE, AF, other, postoperative orthopaedic (70 yrs) 45% male	Exclusions: antiphospholipid antibodies, contraindication for warfarin, previous warfarin, liver disease, renal disease, non-Caucasian, <40 yrs.	Primary Outcome: 1) Feasibility of dosing model Secondary Outcomes: 2) % time INR in-range 3)% pts with INR >4
Anderson (2007) USA	Inpatient & Outpatients	201 with DVT, PE, AF, other, preoperative orthopaedic (61 yrs) 53% male	INR target 2.0-3.0. Exclusions: <18 yrs, women, pregnant, lactating or child-bearing potential, rifampin within 3 wks, co-morbidities precluding standard dosing (advanced physiological age, hepatic or renal insufficiency/creatine of <25 mg/dl, terminal illness)	Primary Outcome 1) % INR out of range/patient Secondary Outcomes: 2)time to INR >3.2 or VitK 3)%pts in range days 5 &8 4)Number of INR measures & dose adjustments 5) %pts with SAEs - INR ≥4, VitK, major bleeding, thromboembolic events, stroke, MI & death)
Caraco (2008) Israel	Inpatients	232 with DVT, PE, AF (58 yrs) 46% male	Exclusions: <18 yrs and baseline INR >1.4	Primary Outcomes: 1) Time to INR >2.0 2) time to stable anticoagulation (defined as 2 INR in-range 7 days apart) Secondary Outcomes: 3) %Time INR in-range 4) Days INR out of range 5) Major bleeding/VTE events

We assessed methodological quality of the included studies for the following components: allocation concealment, randomisation, blinding of outcome assessors, and follow up. Where data was presented only in graphical form we extracted from the figures using the Grab It XP Microsoft excel. http://www.datatrendsoftware.com.

### Data synthesis and analysis

We summarised results as proportion of INRs in range from date of initiation. We reported the proportion of INRs in range at day one through day eight where applicable, as well as the mean time to in range, in days, with standard deviations. For dichotomous outcomes we compared different regimens using relative risks (RR) and calculated 95% confidence intervals (CIs). For continuous variables we compared weighted mean difference (WMD) with 95% CIs. Where we pooled data we used the i-squared statistic to measure statistical heterogeneity for each outcome. Where no heterogeneity was present, we used a fixed-effect meta-analysis and where substantial heterogeneity (i-squared above 50%) was detected, we looked for the direction of effect and used a random effects analysis. No previous protocols have been published for this current review. Insufficient data to pool outcomes by type of loading dose prevented assessment of publication bias and selective reporting of studies.

## Results

**Figure 1:**
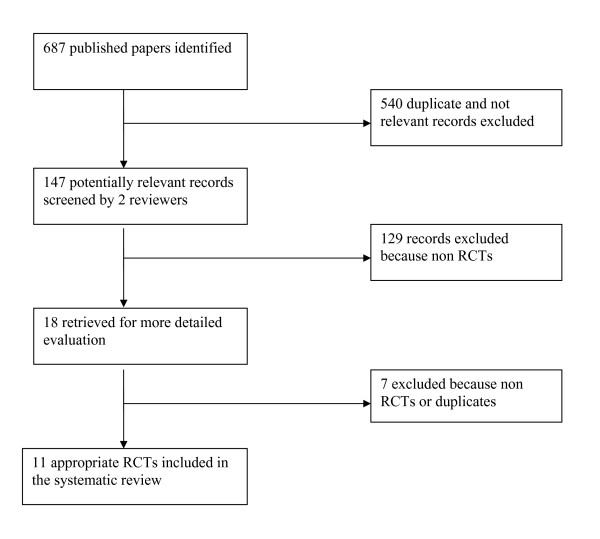
**Flowchart of results**.

We identified 147 potentially relevant records from 687 published papers, of these two reviewers identified 129 as not relevant or not randomized (Figure 1). On analysis of full text papers a further seven were excluded because they were not randomised trials or were duplicate publications. A total of 11 papers met the inclusion criteria [9,11,13,17-24].

All patients included in the studies were newly initiated on warfarin. In ten studies the recruited patients had target INR in the range of 2.0-3.0 [9,13,17-24], in two studies the inclusion criteria were patients with DVT or PE only [22,23]. One further study included patients after heart valve replacement, target INR of 1.5-2.6 [11]. In total 1,340 patients were randomized and eligible (range 38 to 232 participants per study), with 118 (9%) not included in the primary outcome analysis. Reasons being: withdrawn (n = 17), dose deviation or INR missed (n = 68), warfarin stopped or discharged (n = 29) and one each for Vitamin K administered, coincidental bleeding, died, or catheterized. Patients were recruited from both inpatient and outpatient clinics in: USA (4 studies), Canada (3), UK (1), Israel (1), Australia (1) and multinational (1 - Italy & Canada).

Trials varied in relation to the loading doses compared and the dose protocols that were used. (Table 1) The warfarin loading doses compared were: 5 mg versus 10 mg (4 studies) [9,19,22,23]; 5 mg versus 2.5 mg (1) [11]; 5 mg versus dose adjusted for clinical factors (1) [24]; 10 mg versus dose adjusted for age (2) [13,20]; 5 mg versus dose adjusted for genotype (2) [18,21] and 10 mg versus dose adjusted for genotype (1) [17].

Studies used a variety of initiation dosing protocols in both arms of the trials. (Table 2) In six studies INR was measured daily up to day five [9,11,18,19,23,24], and in three studies INR was measured at times pre-specified by the dosing protocol[17,21,22] Follow-up varied from five to 90 days. Outcomes measured included: time to INR in range, percentage time in-range, percentage in-range at day five, percentage with an INR above range, time to reach 'stable' anticoagulation (as defined by each study), percentage with an INR >4.0-5.0, proportion of serious adverse events such as serious bleeding, VTE, death.

**Table 2:** Dosing regimes

Study	Dosing Protocol on Days 1&2 (Reference for nomogram used)
Harrison 1997 ^ξ^	5 mgs on day 1, up-to 5 mgs on day 2 vs. 10 mgs on day 1, up-to 10 mgs on day 2
Crowther 1999 ^ξ^	5 mgs on day 1, up-to 5 mgs on day 2 vs. 10 mgs on day 1, up-to 10 mgs on day 2
Kovacs 2003	5 mgs vs. 10 mgs on days 1&2 ^*δ*^
Quiroz 2006	5 mgs vs. 10 mgs on days 1&2
**5 mg trials**	

Ageno 2001	5 mg Day 0 (subsequent doses adjusted) vs. 2.5 mg on days 0 through 4 (dose modified if <1.5 or >3.0 on day 3)
Shine 2003	5 mg on day 1, up-to 5 mgs on day 2 vs. Calculated dose on day 1, up-to 100% calculated dose on day 2
**Age trials**	

Roberts 1999	Age adjusted nomogram (6-10 mg) on day 1, 0.5-10 mg on day 2 vs. Fennerty protocol (10 mg on day 1, 0.5 mg-10 mg on day 2) ^ψ^
Gedge 2000	Age stratified 65-75 years & 75 yrs - 10 mg on day 1, upto 5 mg on day 2 vs. Modified Fennerty protocol, 10 mg day 1 and up to 10 mgs on day 2
**Genotyping trials**	

Hillman 2005	5 mg on days 1 & 2 vs. Model - genetic nomogram
Anderson 2007	10 mg on days 1 & 2 vs. Model - 2× predicted maintenance dose on days 1 & 2 followed by predicted dose
Caraco 2008	5 mg on day 1 & up to 5 mg on day 2 vs. Model - genetic nomogram

^ξ ^Crowther 1997 report: Crowther MA, Harrison L, Hirsh J. Reply: Warfarin: Less May Be Better. *Ann Intern Med *1997 August 15;127(4):333.

^*δ *^Kovacs MJ, Anderson DA, Wells PS. Prospective assessment of a nomogram for the initiation of oral anticoagulation therapy for outpatient treatment of venous thromboembolism. Pathophysiol Haemost thromb 2002;321:131-133

^ψ ^Fennerty A, Dolben J, Thomas P et al. Flexible induction dose regimen for warfarin and prediction of maintenance dose. *British Medical Journal Clinical Research Ed *1984 April 28;288(6426):1268-70.

Methodological quality was generally poor with only one study (Kovacs 2003) [22] reporting adequate randomization, allocation concealment, double blinding and intention to treat analysis. Study assessment was hindered by lack of reporting of these quality components; however, a number of studies indicate these components did not take place. (Table 3)

**Table 3:** Methodological Quality

	Ageno 2001	Anderson 2007	Caraco 2007	Crowther 1999	Gedge 2000	Harrison 1997	Hillman 2005	Kovacs 2003	Quiroz 2006	Roberts 1999	Shine 2003
Randomisation Method	+	+	+	+	NM	+	+	+	+	NM	+
Concealment of allocation	NM	+	-	NM	NM	NM	+	+	NM	-	+
Double blinding	NM	+	-	-	NM	-	-	+	-	-	NM
Intention to treat	-	-	-	-	-	-	+	+	+	-	-

+ Reported in paper

- Paper indicates that did not take place

NM: Not mentioned in paper

Although there was considerable heterogeneity across the eleven studies in terms of design quality, loading dose protocols, patient population, outcome measures, and length of follow-up (Tables 1,2,3), we grouped them into four clinically relevant categories: 5 mg versus 10 mg [9,19,22,23]; 5 mg versus other doses [11,24]; age adjusted [13,20] and genotype loading dose [17,18,21].

### 5 mg v 10 mg loading dose

**Figure 2:**
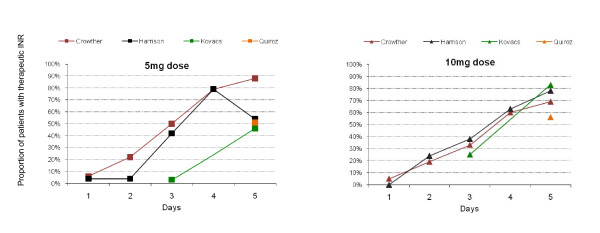
**Proportion of Patients with a therapeutic INR from day of initiation (5 mg vs. 10 mg)**.

**Figure 3:**
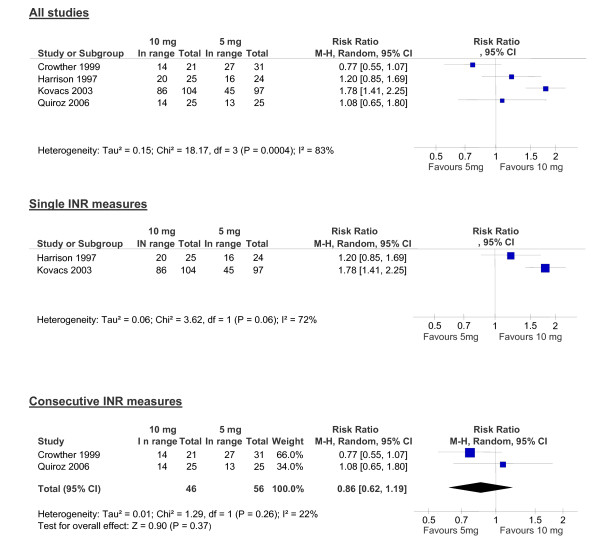
**Proportion of patients with a therapeutic INR at day 5 (10 mg versus 5 mg)**.

Four studies (355 patients) compared 5 mg versus 10 mg loading doses. (Figure 1) [9,19,22,23] Tables 1 and 2 illustrate the nomograms used for dosing which varied across the studies, as well as patient characteristics and inclusion criteria. All four studies reported INR in-range by day five (Figure 2) but high heterogeneity (I^2 ^= 83%) prevented pooling (Figure 3). One possible reason for this is that studies report the proportion either as single or two consecutive INR measures. In the two studies that used single INR measures (Harrison, Kovacs) [9,22] a loading dose of 10 mg led to more patients in range on day five although heterogeneity remained high (Figure 3). Kovacs, also reported the mean time to being in-range was significantly shorter using a 10 mg than a 5 mg loading dose (5.6 vs. 4.2 days, p < 0.001) [22]. (Table 4)

**Table 4:** Summary of primary outcome results (Time to stable INR, supra = therapeutic INR, sub-terapeutic INRs, vitamin K given, and serious adverse events)

		Proportion in INR range									
Study	Dosing	Day 3 (95% CI)	Day 5 95% (CI)	Mean time to time in range Days (SD)	INR ≥4 unless otherwise stated %		Vit K Given N (%)	Serious Adverse Events N (%)	Other primary endpoints		
**5 mg v 10 mg**											
Harrison	5 mg	42%	67%^*δ*^	-	-		1(4%)	0			
	10 mg	36%	80%^*δ*^	-	-		4(16%)	0			
Crowther	5 mg	50% (26 to 75)	88%(75 to 102)	-	-		1(3%)	-	INR 2.0-3.0 for 2 consecutive days & not >3.0;		p < .003
	10 mg	33% (-2 to 68)	69% (39 to 99)	-	-		0	-	10 mg (24%) v 5 mg (66%) RR 2.22 (95% CI 1.3-3.7)		
Kovacs	5 mg	3%*	46% (36 to 57)** P<.001	5.6 (1.4)	INR ≥5 11%		-	2 (2%)			
	10 mg	25%*	83% (74 to 89)**		4.2 (1.1)	9%		-	4 (4%)			
Quiroz	5 mg	-	52%**	Median 5	INR >5 0%		-	0			
	10 mg	-	56%**	Median 5	0%		-	1 (4%)			

		*taken from the published graph ^*δ *^includes discharged with INR in-range from (Ann Int Med Vol 127, 4, pg 133) ** in-range **by **day 5									

**5 mg vs. other doses**											
Ageno	5 mg	-	-	2.0 (1.0)	P < .0001	-	3 (3%)	0	INR > 2.6 5 mg (42%)		p < .05
	2.5 mg	-	-	2.7 (1.2)	P < .0001	-	5 (6%)	0	2.5 mg (26%)		p < .05
Shine	Std (5 mg)	-	63% ^*δ*^	5 (0.9) ^ψ^	P = .007	"high" INR 2%	-	1 (2%)			
	Calc	-	77% ^*δ*^	4.2 (0.9)^ψ^	P = .007	INR >4.4 5%	-	1 (2%)			

		^*δ *^INR within range on or before day 6 ^ψ ^completers only									

**Age trials**											
Roberts	Age adjusted		47%*	3.7 (1.3) ^Φ^	6%		-	0 ^Φ^	INR 2.0 - 3.0 for 2 consecutive days		
	Fennerty		25%*	4.3 (1.2) ^Φ^	32%		-	0 ^Φ^	Age v Fennerty;		p = 0.003
					INR >4.5				Mean days in range (SD)		
Gedge	Age 65-75 yrs	-	-	4.6 (1.6)	P = .03	3% P < .05	0	0	Age	3.0 (1.3)	p = 0.03
	New Fennerty 65-75 yrs	-	-	3.8 (0.8)	P = .03	20% P < .05	^^^	0	New Fennerty	2.7 (1.3)	p = 0.03
					INR >4.5				Mean days in range (SD)		
	Age >75 yrs	-	-	4.5 (1.4)	P = .003	3% P < .01	0	0	Age	2.9 (1.1)	p = 0.04
	New Fennerty >75 yrs	-	-	3.5 (0.7)	P = .003	37% P < .01	^^^	0	New Fennerty	2.4 (1.3)	p = 0.04

		*taken from the published graph ^Φ ^author correspondence ^^ ^1 (age not stated)									

**Genotyping**											
Hillman	Model	-	-	-	33%		0	2 (10%)	% time INR in range 5 mg 42%		
	5 mg	-	-	-	30%		2 (doses)	5 (28%)	Model 42%		
Anderson	Model	-	70%	-	30%		-	4 (4%)	average % of INR outside range 10 mg 33%		
	10 mg	-	68%	-	37%		-	5 (5%)	Model 31%		
									% time INR in range		
Caraco	Model	1% *	49% *	4.8 (1.5)	P < .001	-	0	0	5 mg 25%	P < 0.001	
	STD 5 mg	1% *	11% *	7.5 (3.1)	P < .001	-	1 (1%)	1 (1%)	Model 45%	P < 0.001	
		*taken from the published graph									

In contrast, in the two studies that required two consecutive INRs at day five (Crowther, Quiroz) [19,23] a 10 mg loading dose did not lead to more patients in-range on day five (RR = 0.86, 95% CI, 0.62 to 1.19, p = 0.37, I^2 ^22%). In addition, in the Quiroz study [23] the authors report the 5 mg group achieved therapeutic INRs more often during days 6 to 14 (raw data not available), reporting no difference in median time to two consecutive INR being in-range (Table 4).

Kovacs [22] and Quiroz [23] reported the proportion of patients with INR ≥5.0 (Table 4) and found no significant differences between the two dosing groups. Harrison [9] reported administering Vitamin K more frequently to patients in the 10 mg group; however, the difference was not significant and it was administered to patients with INR ≥4.8. Although the number of serious adverse events was reported by three studies [9,22,23], there was insufficient power to evaluate the effect of the loading doses on these and there was considerable variation in the time frames used - 5 days to 90 days (Table 1).

### 5 mg versus other doses

Two studies (322 patients) compared a 5 mg loading dose with 2.5 mg, (Ageno) [11] and 5 mg with a calculated dose (Shine) [24]. In the Ageno study in heart-valve replacement patients (INR target 1.5 to 2.6), patients receiving 2.5 mg took longer to achieve the therapeutic range (2.7 vs. 2.0 days; p < 0.0001), but were less likely to have an INR >2.6 (26% vs. 42;% p < .05) and had less days with INR >2.6 (average 0.9 vs. 0.45 days; p = 0.003). There were no serious adverse events in either group and although Vitamin K was administered to more patients in the 2.5 mg group, the difference was not significant.

Shine compared 5 mg with a calculated dose [24], which took account of age, weight, serum albumin and active malignancy. Patients receiving the calculated dose achieved the target range quicker (4.2 days vs. 5 days, p = 0.007); but there was no difference in proportion achieving INR in-range on or before day 6: 77% calculated dose compared to 63% in the 5 mg dose (RR = 1.22, 95% CI, 0.88 to 1.70, p = 0.24). There were no differences in serious adverse events or above-range INR (Table 4).

### Age adjusted

Two studies (192 patients) compared loading doses adjusted for age (Roberts, Gedge) [13,20] with Fennerty's protocol [13] or a modified Fennerty protocol [20] in the standard arm (Table 2).

**Figure 4:**
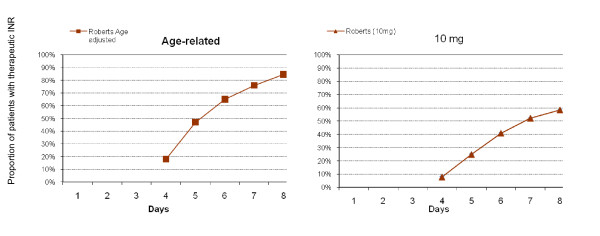
**Proportion of Patients with a therapeutic INR from day of initiation (Age related vs. 10 mg)**.

In the Roberts study, the age adjusted protocol specified different loading doses for five age groups[13] The first dose for each of these groups was: age up to 50 years - 10 mg; 51 to 65 years - 9 mg; 66 to 80 years - 7.5 mg; and >80 years - 6 mg. This dose was either repeated on day two or adjusted according to INR levels. The doses were further decreased by 33% if the patients had one or more of: severe congestive failure, severe chronic obstructive airways disease or amiodarone use. Table 4 shows that by day five Roberts reported more patients in the age adjusted group achieved a stable INR (defined as in-range on 2 consecutive days or within 0.5) than the Fennerty group (48% vs. 22% p = 0.02), and this trend continued throughout the study (Figure 4).

In the Gedge study elderly patients (≥65 yrs) in the age adjusted arm were given 10 mgs on day one and 5 mgs on day two (or less depending on INR levels) [20]. The mean time to an in-range INR was significantly longer for patients on the age adjusted regimen: age 65 to 75 years (4.6 vs. 3.8 days p = 0.03); age >75 years (4.5 vs. 3.5 days p = 0.003). In both studies significantly fewer patients on the age adjusted regimens had high out-of-range INR (Table 4).

### Genotyping trials

**Figure 5:**
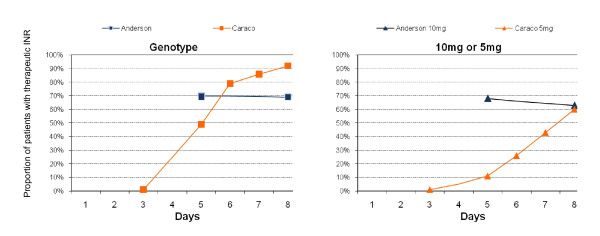
**Proportion of Patients with a therapeutic INR from day of initiation (Genotype vs. 10 mg or 5 mg)**.

Three studies (471 patients) compared loading doses calculated according to patient genotype (genotype model) with 5 mg or 10 mg loading doses[17,18,21] Anderson [17] and Caraco [18] reported a greater proportion of patients' in-range in the genotype group on day five (Table 4). Caraco also reported that the genotype groups spent significantly more time in-range (p < 0.001) but the figure at day five of 15% in range in the 5 mg group was significantly lower than expected at this dose in comparison to similar arms from the other 5 mg trials. (Figure 5) The other two studies report no significant differences between genotype guided, and 5 mg or 10 mg initiation doses. No significant differences were seen for adverse events and no studies were adequately powered to show a difference in major bleeds.

## Discussion

Our systematic review is the first comprehensive analysis of randomized trials of different approaches to the initiation of warfarin. Overall we found a 10 mg loading dose makes a single therapeutic INR measure in-range more likely at five days. Yet, when we analysed two consecutive INR measures at day five the benefits of a 10 mg loading dose were not as apparent. The high heterogeneity between the numbers of patients in range in the 10 mg trials at day five probably reflects that this measure is highly variable and not the best overall measure of the quality of INR control. A 5 mg loading dose compared to 2.5 mg slightly decreased the time to range - about half a day - but at the expense of a greater proportion of overall higher INRs. We found some evidence that age adjusted nomograms may be of benefit in the elderly; however, trials were underpowered to detect important rates of adverse events. The evidence of benefit for genotyping proved disappointing, as in the one trial which showed a significant benefit, the quality of INR control for the comparator (5 mg loading dose) was substantially worse than any other 5 mg study groups in the systematic review. Recent work from a cohort of 4,043 patients which stated the use of pharmacogenetics was appropriate for estimating the initial dose closer to the maintenance dose may have overestimated the potential usefulness of such an approach [25].

Our results are not definitive as trials were generally small; however, they do raise important issues for current practice for the initiation of warfarin. The question as to what is the optimal initiation dose remains unanswered by analysis of the current evidence-base. A 5 mg regimen has been shown to give a more accurate prediction of maintenance dose (correlation co-efficient for predicted versus actual maintenance dose, r = 0.985) [12]. Whether this gives rise to a reduction in adverse events remains unanswered. In the American College of Chest Physicians Guidelines a 5 mg loading dose is potentially appropriate in the elderly patient, in patients with impaired nutrition, liver disease or congestive heart failure and in patients as risk of bleeding [26].

From our systematic review there is little evidence to use genotyping, which conflicts with the recent FDA statement, and the change in labelling for warfarin therapy, which states: "...lower initiation doses should be considered for patients with certain genetic variations in CYP2C9" [27]. Our findings are in accordance with a recent systemic review [28] that showed there are only three randomized trials of genotyping; which are underpowered, and with significant heterogeneity between trials results. In addition, a recent editorial by Ansell notes "most problematic is that the intervention arm of each trial is considerably different" [27]. Therefore current use of genotyping is not underpinned by the evidence and should be discouraged,

During treatment induction with warfarin, elderly patients are especially at high risk of over-anticoagulation [29]. Based on studies that show, generally, daily maintenance doses are about 3 to 4 mg in the elderly [30] the evidence suggests an age adjusted initiation strategy dosage may improve the quality of control. If time is not a crucial issue then an initiation dose of 2.5 mg is a plausible alternative and the evidence suggests there is little difference between 2.5 and 5 mg dose.

A number of limitations are worth noting. Firstly, although our search was comprehensive, the possibility of missing trials exists. We attempted to overcome this by citation searching and snowballing of the literature. Secondly, our conclusions are limited by the quality of the trials, with only one study scoring high on methodological quality. Also the results are not significant, as trials and any pooling of effects are underpowered. A large multicentre trial is currently warranted which should address important adverse event rates by being adequately powered to detect these. Thirdly, often data was missing from the reported studies, and heterogeneity in how primary and secondary outcomes were reported prevented adequate pooling and firm conclusions to be drawn. This can be rectified by standardized reporting which would include single and consecutive measures reported for outcomes at days 3 through to 8, follow up for adverse events for 30 days after initiation and proportion of INR measures ≥4.

## Conclusions

In conclusion our review shows there is a paucity of high quality evidence to guide initiation of warfarin. There is no evidence to suggest a 10 mg loading dose is currently superior to 5 mg. In the elderly lower initiation doses or age adjusted doses may be more appropriate, leading to less higher INRs. Currently there is insufficient evidence to warrant genotype guided initiation, and an adequately powered trial to detect effects on adverse events is currently warranted.

## Competing interests

The authors declare that there are no competing interests.

## Authors' contributions

CH, ST, YW, AW and DK conceived of the study. RP, CB and CH had input to the statistical analyses. ST, CB, AW and YW contributed to the data extraction. All authors contributed to the draft of the manuscript, approved the analyses and read and approved the final manuscript.

## Funding

Dr Carl Heneghan is funded by a NIHR Walport Clinical Lecturer Post. The Department of Primary Health Care at Oxford University is part of the NIHR School of Primary Care research, which provided financial support for Sally Tyndel and Alison Ward. Dr Claire Bankhead, Dr Rafael Perera and Dr Yi Wan received funding from a National Institute of Health Research (NIHR) Health technology project grant on monitoring in chronic disease. Dr David Keeling is funded by the Oxford Radcliffe Hospitals Trust. The funders had no role in the design of the protocol, the analysis or publication of the final manuscript.

## Pre-publication history

The pre-publication history for this paper can be accessed here:

http://www.biomedcentral.com/1471-2261/10/18/prepub
